# Safety and Outcomes of Percutaneous Dilatational Tracheostomy in Patients with Hematologic Malignancies: A Retrospective Cohort Study

**DOI:** 10.3390/jcm14020657

**Published:** 2025-01-20

**Authors:** Asaf Miller, Roee Noy, Omri Simchon, Natalia Gvozdev, Yotam Shkedy, Danny Epstein

**Affiliations:** 1 Medical Intensive Care Unit, Rambam Health Care Campus, HaAliya HaShniya St 8, Haifa 3109601, Israel; 2 Department of Otolaryngology-Head and Neck Surgery, Rambam Health Care Campus, HaAliya HaShniya St 8, Haifa 3109601, Israel; 3 Ruth and Bruce Rappaport Faculty of Medicine, Technion, 1 Efron St. Bat Galim, Haifa 3525433, Israel; 4 Department of Anesthesiology, Rambam Health Care Campus, HaAliya HaShniya St 8, Haifa 3109601, Israel; 5 Critical Care Division, Rambam Health Care Campus, HaAliya HaShniya St 8, Haifa 3109601, Israel

**Keywords:** tracheostomy, complications, hemorrhage, percutaneous, dilatational, intensive care unit, hematological malignancies

## Abstract

**Background/Objectives**: Patients with hematologic malignancy (HM) often experience high rates of thrombocytopenia, thrombocytopathy, anemia, leukopenia, and coagulopathy, which can significantly increase the risk of procedural and postoperative complications. This study aimed to evaluate the safety and outcomes of percutaneous dilatational tracheostomy (PDT) in critically ill patients with HM. **Methods**: This retrospective cohort study included patients with HM who underwent PDT between 2012 and 2023 at a tertiary academic center. The primary outcome was early (7-day) bleeding complications rate. Secondary outcomes included PDT-related mortality, and mortality at 1 week, 30 days, and 1 year. Analyses were performed using a propensity-matched cohort to ensure balanced comparisons between groups. **Results**: Of the 1627 patients included in the analysis, 65 (4%) had HM. Patients with HM had a significantly higher Charlson comorbidity index and exhibited significantly higher rates of thrombocytopenia (platelet count < 100,000/mcL) compared to those without HM (8.0 [IQR 5.0–11.3] vs. 5.0 [IQR 2.0–7.0], *p* < 0.001; and 49.2% vs. 5.0%, *p* < 0.001, respectively). After propensity score matching, the one-week mortality rate was significantly higher in the HM group (23.4% vs. 4.3%, *p* = 0.007). However, the rates of intraoperative and bleeding complications as well as one-year mortality rates were similar between the groups. **Conclusions**: PDT can be safely performed in critically ill patients with HM. However, these patients exhibit high early mortality rates following the procedure.

## 1. Introduction

Hematologic malignancy (HM) comprise a diverse group of conditions arising from cells in the bone marrow and lymphatic system. In the United States alone, approximately 1.4 million people are living with or in remission from HM. In 2019, an estimated 176,200 new cases of HM were diagnosed, accounting for 10% of all new cancer cases [1,2,3].

The treatment of hematologic malignancies has undergone significant changes in recent years [4]. Recent advancements, combined with improvements in supportive care, have enhanced survival rates for patients with HM. However, these developments have also resulted in a growing number of patients vulnerable to life-threatening complications, necessitating admission to an intensive care unit (ICU) [5,6,7]. Approximately 14% of patients with HM are admitted to the ICU within the first year after diagnosis [7]. Historically, ICU mortality in this cohort has been reported to reach as high as 90% [8,9,10]. However, more recent data from high-volume centers indicate an improvement, with rates dropping to 50% or lower [8].

Acute respiratory failure is a common and life-threatening complication in patients with HMs. Among those requiring ICU admission, respiratory failure accounts for 60% to 80% of cases [11]. Acute respiratory failure is the most common reason for ICU admission, occurring in 10–20% of patients with lymphoma or leukemia and approximately 50% of those with neutropenia or following bone marrow transplantation [12,13]. This condition is associated with particularly high mortality rates, especially among mechanically ventilated patients and recipients of allogeneic bone marrow transplants [14,15]. While nearly half succumb to their illness, there has been a trend towards improved survival in recent years [16]. However, some patients recovering from their acute illness may develop chronic respiratory failure, resulting from either muscle weakness or lung disease. This group may require prolonged mechanical ventilation.

Tracheostomy is performed in 10–20% of patients requiring prolonged mechanical ventilation, with its prevalence increasing over the past two decades [17,18]. Although the proportion of patients with HM requiring tracheostomy has not been specifically evaluated, it is anticipated to be higher than in the general population of critically ill patients with respiratory failure. This is because HM has been identified as a risk factor for prolonged weaning and the need for tracheostomy [19].

Percutaneous dilatational tracheostomy (PDT) was first performed in 1955 [20]. In recent years, it has become the preferred procedure for critically ill patients requiring prolonged respiratory support. PDT has largely replaced conventional surgical tracheostomy in ICU patients, offering several advantages, including ease of performance, fewer complications, the elimination of additional transportation requirements, and greater cost-effectiveness. Furthermore, as PDT is performed at the bedside, it avoids the inconvenience of long waiting lists for operating room scheduling, significantly reducing the delay between the decision to perform a tracheostomy and the actual procedure [21]. PDT was shown to be safe in high-risk surgical patients, such as those suffering from morbid obesity, coagulopathy, cirrhosis, and those treated with dual antiplatelets [22,23,24,25,26]. Patients with HM often experience high rates of thrombocytopenia, anemia, leukopenia, and coagulopathy, increasing their risk of procedural and postoperative complications, such as bleeding, airway injury, and surgical site infections [27].

Currently, there are no data comparing the safety and complication rates of PDT in these patients to those without hematologic diseases. The aim of our study was to evaluate the safety and bleeding risk of PDT in critically ill patients with HM. We hypothesized that patients with HM might exhibit distinct outcomes compared to other critically ill patients, owing to their unique baseline characteristics and elevated risk of bleeding.

## 2. Materials and Methods

### 2.1. Study Design and Settings

This retrospective cohort study received approval from the Institutional Review Board of Rambam Health Care Center in Haifa, Israel, with the requirement for informed consent being waived (approval number: 0143-21-RMB).

We conducted a chart review to identify critically ill patients who underwent PDT between January 2012 and March 2023. Rambam Health Care Center is a 1000-bed tertiary academic hospital and the sole Level I trauma and burn center in the region, serving a population of over two million. The hospital includes medical, surgical, burn, neurosurgical, and cardiothoracic intensive care units, along with six intermediate care units.

Only elective procedures indicated for prolonged mechanical ventilation were included in the study. Patients needing emergency tracheostomy or undergoing tracheostomy for maxillofacial trauma or obstructing tumors were excluded.

The study group included all patients with an active diagnosis of HM, as defined by ICD-10 and the Hematological Malignancy Research Network, confirmed during the chart review. Patients were categorized by expected survival rates into three groups: good survival (>70% 5-year survival), medium survival (30–70%), and poor survival (<30%) [28]. For patients without HM, the absence of the disease was also confirmed during the chart review. These patients served as a control group.

### 2.2. PDT Procedure

During the study period, all PDTs were performed by fully trained intensivists, otolaryngologists, or thoracic surgeons. Consistent with guidelines from the American Thoracic Society, the European Respiratory Society, and the American College of Chest Physicians, all physicians had prior experience with at least 30 PDTs [29,30].

All PDTs were performed at the bedside using the modified Ciaglia technique with either Blue Rhino (Cook Critical Care, Bloomington, IN, USA) or Portex Ultraperc™ (Smith Medical, Hythe, Kent, UK) kits, along with 7.5 or 8 mm cuffed tracheostomy tubes [21]. Following adequate sedation and analgesia with intravenous propofol (1.5 mg/kg) or midazolam (5–10 mg) and fentanyl (50–100 μg), neuromuscular blockade with rocuronium (0.6–1.2 mg/kg) was administered at the anesthesiologist’s discretion. Patients were positioned supine with a rolled-up towel under the shoulders to achieve neck hyperextension, while those with cervical spine injuries were maintained in a spine-neutral position. Ventilation was provided in a volume control mode with an FiO_2_ of 1.0 and a positive end-expiratory pressure (PEEP) of 5 cmH_2_O.

After local infiltration with 2% lidocaine, a 10 mm midline incision was made over the trachea at the level of the second tracheal ring. The subcutaneous tissue was dissected to the trachea using a hemostat clamp, and tracheal rings were identified by palpation. Under direct visualization with a laryngoscope, the tracheal tube was retracted until the cuff was above the vocal cords.

A 14-gauge catheter introducer needle was used to puncture the trachea, and intraluminal placement was confirmed by air aspiration through a saline-filled syringe. Using the Seldinger technique, a J-tipped guidewire was introduced through the catheter, which was subsequently removed. The trachea was initially dilated with a 14 Fr introducer dilator, followed by further dilation using an appropriate-sized dilator. A preloaded cuffed tracheostomy tube was then advanced over the introducer and placed.

Correct placement of the tracheostomy tube was verified by chest auscultation and end-tidal CO_2_ (EtCO_2_) monitoring. The tracheostomy was secured with sutures and a tie, and a routine chest radiograph was performed. Real-time ultrasound and bronchoscopy guidance were employed at the discretion of the attending physician in cases where PDT was anticipated to be challenging.

Medical thromboprophylaxis with low-molecular-weight heparin or unfractionated heparin was paused for 12 h, while therapeutic anticoagulation was withheld for at least 24 h. Thromboprophylaxis was resumed 12 h post-procedure and therapeutic anticoagulation after 24 h. Patients with a platelet count below 50,000/μL or an INR greater than 1.5 received platelets or fresh frozen plasma (FFP), respectively, immediately before the procedure at the discretion of the attending physician. Patients on dual antiplatelet therapy were excluded from the current study.

### 2.3. Outcome Measures and Variables

The primary outcome of this study was early (7-day) bleeding, categorized as either minor (requiring direct pressure, dressing change, or bedside sutures) or major (requiring exploration in an operating room, urgent therapeutic bronchoscopy due to airway compromise, a hemoglobin decrease of ≥2 g/dL, or transfusion of ≥2 units of packed red cells) [31,32]. Secondary outcomes included PDT-related mortality, 7-day, 30-day, and 1-year all-cause mortality.

The following demographic and clinical data were retrieved from the patients’ electronic medical records: age, sex, body mass index (BMI), Charlson Comorbidity Index, admission diagnosis, duration of mechanical ventilation prior to tracheostomy, periprocedural use of antiplatelet agents, platelet count, partial thromboplastin time (PTT), and international normalized ratio (INR) within 24–48 h before the procedure. Additionally, tracheostomy-related mortality, as well as 7-day, 30-day, and 1-year all-cause mortality, were documented. The Charlson Comorbidity Index is a widely used tool for predicting mortality risk by accounting for a patient’s burden of comorbid conditions. Each comorbidity is assigned a weighted score based on its severity and potential impact on survival, and the total score reflects the cumulative effect of all comorbidities. Higher scores are associated with worse outcomes, including increased short-term and long-term mortality [33].

A chart review was conducted in accordance with established methods for retrospective studies [34]. Using a structured form, abstractors familiar with the study objectives and hypothesis independently reviewed all charts. A standardized data collection tool was employed to systematically extract information from medical records. Three abstractors collected the independent variables, while a separate investigator recorded the outcomes. The STrengthening the Reporting of OBservational Studies in Epidemiology (STROBE) statement was followed to guide the reporting of this research [35].

### 2.4. Statistical Analysis

Categorical variables were reported as frequencies and percentages, while continuous variables were presented as medians with an interquartile range (IQR). We used the Mann–Whitney U test for continuous parameters and the χ^2^ test for categorical variables. A *p*-value of less than 0.05 was considered statistically significant. All available data from our databases within the study time frame were used. Missing data were addressed using listwise deletion. Propensity score matching was performed to adjust for imbalances in covariates between the groups (admission diagnosis, Charlson comorbidity index, and platelet count). We defined the allowable caliper width as equal to 0.2 of the pooled standard deviation of the logit of the propensity score. Analysis was conducted using IBM SPSS Statistics for Windows, Version 26.0 (IBM Corp., Armonk, NY, USA, 2019).

## 3. Results

**Figure 1:**
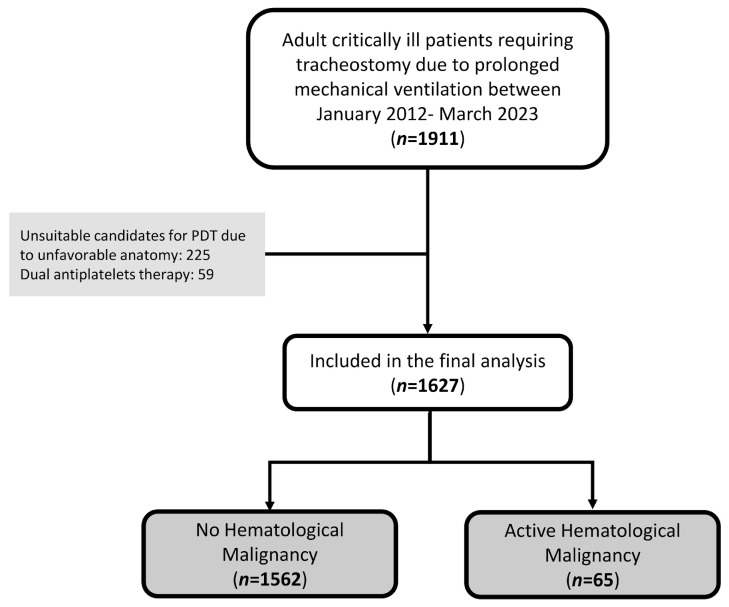
Study flow chart.

During the study period, a total of 1911 patients required tracheostomy due to prolonged mechanical ventilation. Two hundred and twenty-five patients (11.8%) were deemed unsuitable candidates for PDT due to unfavorable anatomy identified during the initial clinical evaluation, such as having thick, short necks; morbid obesity; cervical tumor; or goiter. These patients underwent surgical tracheostomy. An additional total of 59 patients (3.1%) were excluded due to dual antiplatelet therapy. A total of 1627 patients were included in the analysis, of whom 65 (4.0%) had active HM, while 1562 (96.0%) comprised the control group (Figure 1).

The characteristics of the hematologic diseases of the patients included in the study are described in Table 1.

Patients in both study groups had comparable characteristics; however, those with HM had significantly higher Charlson comorbidity index scores (8.0, IQR 5.0–11.3 vs. 5.0, IQR 2.0–7.0, *p* < 0.001) and a higher prevalence of thrombocytopenia (platelet count < 100,000/mcL) (49.2% vs. 5.0%, *p* < 0.001). The admission diagnoses also differed between the groups (Table 2). One patient in the hematological group received FFP transfusion before the procedure. In both groups, most patients underwent late tracheostomy (≥10 days after the initiation of invasive mechanical ventilation). Early tracheostomy was performed in 26.2% of patients with HM and 28.0% of patients without hematologic disease (*p* = 0.74).

In the HM group, five patients (7.7%) developed early bleeding, all of which were minor. In comparison, the control group experienced 80 bleeding events (5.1%), consisting of 75 minor and 5 major bleedings (*p* = 0.36). There was a single case of PDT-related mortality among the patients with HM (1.5%), compared to nine cases (0.6%) in the control group (*p* = 0.33). However, 1-week, 30-day, and 1-year mortality rates were significantly higher among the patients with HM.

After incorporating the propensity score and matching for admission diagnosis, Charlson comorbidity index, and platelet count, only the one-week mortality rates were significantly higher among patients with hematological disease (23.4% vs. 4.3%, *p* = 0.007). The rates of bleeding complications and one-year mortality rates were comparable between the groups (Table 3).

## 4. Discussion

In this study, we found that PDT can be safely performed in critically ill patients with HM requiring prolonged ventilatory support. After adjusting for the severity of the comorbidities and other confounders, our analysis showed that PDT was not associated with higher rates of early bleeding in this population. However, patients with HMs exhibited higher short-term mortality rates at 7 and 30 days, though these outcomes were not directly attributable to the procedure. There were no significant differences in 1-year all-cause mortality rates.

Patients with HMs admitted to the ICU have high mortality rates, and the development of respiratory failure requiring invasive mechanical ventilation further increases this risk [36]. The treatment of respiratory failure in this unique population is challenging. Effective ICU management as a bridge to curative anticancer treatment requires close collaboration among hematologists, intensivists, chest physicians, infectious disease specialists, clinical microbiologists, and other interdisciplinary healthcare professionals [11]. A recent large-scale worldwide study conducted by Bris et al. found a 64.4% 90-day mortality rate among patients with HM admitted to the ICU due to acute respiratory failure [37]. The clinical course of these critically ill patients is marked by a variety of complications, including a high prevalence of infections and sepsis, metabolic disturbances, and an increased risk of bleeding and thrombotic events [27]. Additionally, these patients experience higher complication rates and elevated mortality following various surgical interventions, including minor ones [38,39,40]. Forrester et al. found that patients with HM have a standardized risk ratio of 2.9 (IQR 2.2–3.8) for death following most general surgery procedures [38]. In another study conducted by Nguyen et al., patients with HM experienced significantly higher rates of postoperative complications following cardiac surgery, including a greater need for blood component transfusions, respiratory complications, acute renal failure, and prolonged ICU and hospital stays. Thirty-day mortality was also numerically higher in these patients [39,41,42].

While some complications of surgical procedures may be attributed to immunosuppression caused by either the disease itself or the prescribed therapy, immediate complications are typically due to the increased risk of bleeding, which is common in this population [43]. Although the bleeding tendency in some patients can be explained by thrombocytopenia or prolonged prothrombin time, reliable biomarkers to accurately assess bleeding risks in critically ill patients with HMs are still lacking [43]. Vigneron et al. reported that, among 1012 critically ill hematologic patients, 10.8% experienced severe bleeding events, classified as grade 3 or 4 according to the World Health Organization (WHO). The authors focused solely on ICU-acquired hemorrhages, which were defined as bleeding events occurring at least 24 h after ICU admission. Of the severe bleeding events, 57.8% were classified as grade 4, indicating potentially life-threatening hemorrhage. Additionally, patients who experienced bleeding events had significantly higher ICU mortality, longer ICU stays, and a greater need for supportive treatments, including mechanical ventilation, vasopressors, and renal replacement therapy, as well as higher transfusion requirements [44].

Limited information is available on the safety and outcomes of tracheostomy in cancer patients, particularly those with HM. Angelberger et al. evaluated the safety of PDT and surgical tracheostomy in immunocompromised patients with hematological diseases, most of whom had low platelet counts [45]. In this retrospective cohort study of 84 patients, including 63 who underwent PDT, periprocedural complications occurred in three cases (4.6%), none of which were fatal. Postprocedural bleeding was reported in two patients (3.2%). Notably, bleeding was more common among patients undergoing the open procedure. Kumar et al. reported that tracheostomy can be safely performed in critically ill cancer patients, including those with significant comorbidities, thrombocytopenia, and COVID-19 infection. However, a high short-term mortality rate was observed in this population. The majority of the patients included in the study had solid tumors and only 36% had HM. There were no major adverse events or deaths during the procedure. At a 6-month follow-up, 70% of patients had died [46]. Previous studies have shown that PDT can be safely performed in other critically ill patients at high risk of bleeding, such as those with cirrhosis or receiving dual antiplatelet therapy [24,25]. In a small-scale study of 26 neutropenic patients, tracheostomy was also found to be safe [47].

Our study revealed a low complication rate among patients with HMs undergoing PDT. Specifically, there was only one case of periprocedural mortality (1.5%) and five cases of minor early bleeding (7.7%). However, up to 20% of these patients died within one week of the procedure. Long-term mortality rates after one year were also high but comparable to those observed in patients without HMs. The high rates of early mortality may be attributed to the severity of the critical illness itself; however, other factors, such as inappropriate timing of the procedure or an increased risk of infections following PDT, should also be considered and evaluated. The hematologic patients included in our study underwent tracheostomy after a median of 12 days of invasive mechanical ventilation, with only a quarter of them undergoing early tracheostomy, typically defined in the literature as a procedure performed within 7–10 days of invasive mechanical ventilation. Numerous studies have shown that early tracheostomy is associated with modestly decreased mortality compared to late tracheostomy, as well as reduced ICU length of stay, shorter duration of mechanical ventilation, and lower rates of pulmonary infections [48,49]. Earlier tracheostomy is linked to a quicker attainment of patient-centered outcomes, including the ability to resume daily activities such as talking, out-of-bed mobility, and eating/drinking. Additionally, these patients require fewer sedatives and analgesics [50]. Certain patient groups may benefit more from early tracheostomy than others. These include patients with severe traumatic brain injury, stroke, acute traumatic spinal cord injury, multiple rib fractures, and possibly those with severe polytrauma [41,42,51,52,53]. A study of Blot et al. published in 1995 found that among mechanically ventilated neutropenic patents, tracheostomy performed within 48 h from initiation of mechanical ventilation was associated with a trend towards longer survival [54]. The timing of tracheostomy has not been specifically evaluated in cancer patients or in those with HM. Given that these patients may have lower chances of successful weaning from mechanical ventilation [19], we speculate that early tracheostomy should be considered for this unique population. It may be associated with improved outcomes, as it allows for a gradual reduction in the continuous sedative infusion and inotropic infusion agents used to counteract arterial hypotension. Studies suggest that propofol, remifentanil, midazolam, and muscle relaxants may contribute to immunodepression, prolonged release of cytokines, micro-aspiration, abnormal peristalsis, microcirculatory effects, and predisposition to infections [55]. Early tracheostomy may accelerate the weaning process, reduce the risk of ventilator-associated pneumonia and systemic infections, accelerate the patient’s awakening and motility, and facilitate nursing care, thereby enhancing the opportunity for early rehabilitation and discharge [56].

Our study had several limitations. First, its retrospective design carries inherent disadvantages. Second, the small size of the HM group may have introduced bias into the results. Third, our analysis focused solely on bleeding complications and did not address other potential complications, such as the prevalence of ventilator-associated pneumonia or tracheostomy stoma infections following the procedure.

## 5. Conclusions

In conclusion, PDT is not associated with higher rates of early bleeding or procedure-related mortality in critically ill patients with HM. However, approximately one in four patients undergoing this procedure is expected to die within the first week. Further studies are needed to validate these findings and explore potential risk factors contributing to early mortality in this unique population. The timing of tracheostomy and its association with clinical outcomes should be more thoroughly evaluated.

## Figures and Tables

**Table 1:** Types of hematological malignancy included in the study (*n* = 65) grouped by survival rates based on the Hematological Malignancies Research Network [8].

Prognosis	Number of Patents (%)
Good survival rate (5-year survival >70%)	15 (23.1%)
Medium survival rate (5-year survival 30–70%)	32 (49.2%)
Poor survival rate (<30% 5-year survival)	18 (27.7%)

**Table 2:** Clinical and laboratory characteristics of the study cohort (*n* = 1627).

		**No Hematologic Malignancy** **(*n* = 1562)**	**Active Hematologic Malignancy** **(*n* = 65)**	***p*-Value**
Age, years, median (IQR)		64.1 (49.2–74.0)	64.9 (55.9–70.1)	0.97
Male gender, *n* (%)		1045 (66.9%)	40 (61.5%)	0.37
Charlson comorbidity index, median (IQR) ^a^		5.0 (2.0–7.0)	8.0 (5.0–11.3)	***<0.001***
Admission diagnosis				
	Medical, *n* (%)	618 (39.6%)	41 (63.1%)	***<0.001***
	Neurological, *n* (%)	254 (16.3%)	5 (7.7%)	
	Surgical, *n* (%)	103 (6.6%)	4 (6.2%)	
	Trauma, *n* (%)	400 (25.6%)	2 (3.1%)	
	Other, *n* (%)	186 (11.9%)	13 (20.0%)	
BMI kg/m^2^, median (IQR)		26.0 (23.11–30.75)	26.0 (22.00–29.19)	0.07
Mechanical ventilation duration before tracheostomy, median (IQR) ^b^		12.0 (9.0–17.0)	12.0 (9.0–15.0)	0.26
Early tracheostomy (<10 days of mechanical ventilation prior to PDT), *n* (%)		438 (28.0%)	17 (26.2%)	0.74
Aspirin treatment within 5 days before procedure, *n* (%)		211 (13.5%)	6 (9.2%)	0.32
Clopidogrel treatment within 5 days before procedure, *n* (%)		23 (1.5%)	0 (0.0%)	0.32
Platelets count, K/mcL, median (IQR)		282.0 (197.0–402.0)	100.0 (50.8–242.3)	***<0.001***
Thrombocytopenia, *n* (%) ^c^		78 (5.0%)	32 (49.2%)	***<0.001***
INR, median (IQR)		1.07 (0.99–1.16)	1.07 (1.01–1.19)	0.88
aPTT, sec, median (IQR)		28.7 (26.1–31.5)	27.7 (25.5–31.1)	0.38
Intraoperative complications, *n* (%)		23 (1.5%)	0 (0.0%)	0.32
Early bleeding				
	Minor, *n* (%)	75 (4.8%)	5 (7.7%)	0.29
	Major, *n* (%)	5 (0.3%)	0 (0.0%)	0.65
Late bleeding, *n* (%)		4 (0.3%)	0 (0.0%)	0.68
PDT-related mortality, *n* (%)		9 (0.6%)	1 (1.5%)	0.33
7-day all-cause mortality, *n* (%)		85 (5.4%)	13 (20.0%)	***<0.001***
30-day all-cause mortality, *n* (%)		363 (23.3%)	32 (49.2%)	***<0.001***
1-year all-cause mortality, *n* (%)		765 (49.0%)	48 (73.8%)	***<0.001***

^a^ Charlson comorbidity index was missing in 10 patients (0.6%) in the control group. ^b^ Mechanical ventilation duration was missing in 22 patients (1.4%) in the control group and 1 patient in the active hematological malignancy group (1.5%). ^c^ Thrombocytopenia was defined as platelet count <100 K/mcL. BMI—Body Mass Index; INR—International Normalized Ratio; PTT—Activated Partial Thromboplastin Time; PDT—Percutaneous Dilatational Tracheostomy. Statistically significant values (*p* ≤ 0.05) are given in bold and italics.

**Table 3:** Clinical and laboratory characteristics of the matched cohort.

		**No Hematologic Malignancy** **(*n* = 47)**	**Active Hematologic Malignancy** **(*n* = 47)**	***p*-Value**
Age, years, median (IQR)		65.1 (59.7–72.3)	66.2 (58.8–71.9)	0.89
Male gender, *n* (%)		23 (48.9%)	26 (55.3%)	0.54
Charlson comorbidity index, median (IQR)		7.0 (5.0–9.0)	7.0 (6.0–8.0)	0.58
Admission diagnosis				
	Medical, *n* (%)	32 (68.1%)	32 (68.1%)	1.00
	Neurological, *n* (%)	4 (8.5%)	4 (8.5%)	
	Surgical, *n* (%)	2 (4.3%)	2 (4.3%)	
	Trauma, *n* (%)	1 (2.1%)	1 (2.1%)	
	Other, *n* (%)	8 (17.0%)	8 (17.0%)	
BMI kg/m^2^, median (IQR)		25.0 (23.5–31.0)	25.9 (22.9–29.4)	0.58
Mechanical ventilation duration before tracheostomy, median (IQR)		15.0 (10.3–22.8)	12.0 (10.0–15.0)	***0.01***
Aspirin treatment within 5 days before procedure, *n* (%)		10 (21.3%)	4 (8.5%)	0.08
Clopidogrel treatment within 5 days before procedure, *n* (%)		0 (0.0%)	0 (0.0%)	1.00
Platelets count, mcL, median (IQR)		188.0 (73.0–268.3)	121 (81.0–244.8)	0.34
Thrombocytopenia, *n* (%)		19 (40.4%)	19 (40.4%)	1.00
INR, median (IQR)		1.05 (0.97–1.13)	1.07 (1.02–1.20)	0.31
aPTT, sec, median (IQR)		27.9 (24.2–30.5)	29.3 (26.0–31.8)	0.16
Intraoperative complications, *n* (%)		1 (2.1%)	0 (0.0%)	0.32
Early bleeding				
	Minor, *n* (%)	4 (8.5%)	3 (6.4%)	0.69
	Major, *n* (%)	0 (0.0%)	0 (0.0%)	1.00
Late bleeding, *n* (%)		0 (0.0%)	0 (0.0%)	1.00
PDT-related mortality, *n* (%)		0 (0.0%)	0 (0.0%)	1.00
7-day all-cause mortality, *n* (%)		2 (4.3%)	11 (23.4%)	***0.007***
30-day all-cause mortality, *n* (%)		14 (29.8%)	23 (48.9%)	0.06
1-year all-cause mortality, *n* (%)		38 (80.9%)	35 (74.5%)	0.46

BMI—Body Mass Index; INR- International Normalized Ratio; PTT—Activated Partial Thromboplastin Time; PDT—Percutaneous Dilatational Tracheostomy. Statistically significant values (*p* < 0.05) are given in bold and italics.

## Data Availability

The datasets used and/or analyzed during the current study are available from the corresponding author on reasonable request.
